# Comparison of release kinetics of different cardiac biomarkers in patients undergoing off pump coronary artery bypass surgery and valve replacement surgery for rheumatic heart disease

**DOI:** 10.1186/1749-8090-10-S1-A208

**Published:** 2015-12-16

**Authors:** Surendra K Agarwal, Aditya Kapoor, Shantanu Pande, Gauranga Majumdar, Satyajit Singh, Archana Sinha, Sudeep Kumar, Satyendra Tewari, Naveen Garg, Pravin K Goel

**Affiliations:** 1 Department of CVTS, Sanjay Gandhi Postgraduate Institute of Medical Sciences, Lucknow, 226014, India; 2 Cardiology, Sanjay Gandhi Postgraduate Institute of Medical Sciences, Lucknow, 226014, India

## Background/Introduction

Cardiac biomarkers are released during cardiac surgery. The release kinetics have not been compared in patients undergoing different types of cardiac surgery.

## Aims/Objectives

We compared the release kinetics of BNP, Troponin-I (Tn-I) and CK-MB in patients undergoing off pump CABG (OPCABG) and valve replacement (VR).

## Method

Cardiac biomarkers were serially measured 24 hours prior and 6, 24, 48 hours and 1 month following OPCABG (n = 80, mean age 59.1 years) and VR (n = 50, mean age 36.7 years).

## Results

Mean baseline BNP was significantly higher in VR (304.01 vs 105.8 pg/ml, p < 0.001), although baseline LVEF (54.4% vs 52.5%) was similar in both. Pre-operative Tn-I and CK-MB levels were higher in those undergoing OPCABG. Biomarker release kinetics were significantly different amongst two groups. All three biomarkers increased rapidly within 6 hours of OPCABG. In VR group, BNP levels initially decreased (within 6 hours), and then peaked at 24 hours; in contrast Tn-I and CKMB levels increased within 6 hours, with declining trends at 24 hours. Peak BNP levels occurred usually by 24-48 hours. At 1 month, levels of all biomarkers were similar to baseline.

In patients undergoing OPCABG, those with higher baseline BNP had lower LVEF; while in patients undergoing VR, those with higher baseline BNP levels had higher prevalence of atrial fibrillation and higher RV systolic pressure. Patients with higher pre-operative BNP had longer post-operative inotrope duration and ventilator time. In patients undergoing VR, patients with higher baseline BNP also had longer post-operative ICU and hospital stay. In both groups, baseline, post-operative and delta BNP levels were significant predictors of post-operative inotrope duration and ventilation time.

## Discussion/Conclusion

Patients of RHD undergoing VR had higher baseline BNP levels as compared to those undergoing OPCABG, despite similar LVEF. Release kinetics of biomarkers following OPCABG and valve surgery are significantly different from each other. Of all studied biomarkers, only BNP predicted post-operative inotrope duration and ventilation times.

